# The Combined Treatment of Chinese Herbal Medicines Is Correlated with a Lower Risk of Rheumatoid Arthritis in Patients with Depression: Evidence from a Population-Based Patient–Control Study

**DOI:** 10.3390/ph18040480

**Published:** 2025-03-27

**Authors:** Chieh-Tsung Yen, Hanoch Livneh, Hui-Ju Huang, Ming-Chi Lu, Wei-Jen Chen, Tzung-Yi Tsai

**Affiliations:** 1 Department of Neurology, Dalin Tzu Chi Hospital, Buddhist Tzu Chi Medical Foundation, Chiayi 62247, Taiwan; 2 Rehabilitation Counseling Program, Portland State University, Portland, OR 97207-0751, USA; 3 Department of Nursing, Dalin Tzu Chi Hospital, Buddhist Tzu Chi Medical Foundation, Chiayi 62247, Taiwan; 4 School of Medicine, Tzu Chi University, Hualien 97004, Taiwan; 5 Division of Allergy, Immunology and Rheumatology, Dalin Tzu Chi Hospital, Buddhist Tzu Chi Medical Foundation, Dalin Township, Chiayi 62247, Taiwan; 6 Department of Chinese Medicine, Dalin Tzu Chi Hospital, Buddhist Tzu Chi Medical Foundation, Chiayi 62247, Taiwan; 7 Graduate Institute of Sports Science, National Taiwan Sport University, Taoyuan 333325, Taiwan; 8 School of Post-Baccalaureate Chinese Medicine, Tzu Chi University, Hualien 97004, Taiwan; 9 Center of Sports Medicine, Dalin Tzu Chi Hospital, Buddhist Tzu Chi Medical Foundation, Chiayi 62247, Taiwan; 10 Department of Medical Research, Dalin Tzu Chi Hospital, Buddhist Tzu Chi Medical Foundation, Chiayi 62247, Taiwan; 11 Department of Environmental and Occupational Health, College of Medicine, National Cheng Kung University, Tainan 70428, Taiwan

**Keywords:** depression, rheumatoid arthritis, Chinese herbal medicines, nested patient–control study

## Abstract

**Background:** Major depression places psychological strain on the individual that may increase the risk of developing rheumatoid arthritis (RA). Though the use of Chinese herbal medicines (CHMs) is widespread in clinical practice, its effect on the prevention of RA incidents is still unknown. This study aimed to evaluate the association between CHMs use by patients with depression and their subsequent risk of being diagnosed with RA. **Methods:** This nested case–control study used claims data from a nationwide insurance database. We identified patients aged 20–70 years with newly diagnosed depression and without pre-existing RA between 2002 and 2010. We enrolled those with RA onset occurring after depression by the end of 2013 (*n* = 973). Randomly matched controls were selected from the remaining patients with depression but without RA (*n* = 1946). Conditional logistic regression analysis was executed to assess the association between CHMs use and RA onset. Data are presented as *p*-values with the significance set at 0.05 and as odds ratios (ORs) with 95% confidence intervals (CIs). **Results:** In this study, we found that adding CHMs treatment to conventional antidepressants greatly decreased the subsequent risk of RA among patients with depression, with an ORs of 0.64 (95% CIs: 0.57–0.76). Those using CHMs for more than three years had the most striking benefit, with a 61% lower risk of RA. Notably, initiating CHMs within the first 2 years after depression onset resulted in the greatest decrease in the RA risk. **Conclusion:** Using CHMs with conventional antidepressant therapy reduced the RA risk among patients with depression. Further well-designed randomized controlled trials are needed to determine the molecular mechanism underlying the action of these herbal agents.

## 1. Introduction

Compelling evidence suggests that chronic inflammation plays a pivotal role in many pathophysiological conditions, thereby negatively striking human health, most notably affective disorders, such as depression [1]. According to a report on the global disease burden by the World Health Organization (WHO), depressive disorder posed the second-highest burden of all diseases in 2020 and is expected to become the world’s largest disease burden by 2030 [2]. In the United States, a nationwide survey showed that approximately 19 million adults (about 6% of the population) experienced a major depressive episode during 2020, with nearly one in five adults having a diagnosis of depression [3]. One recent report showed that the economic burden of major depressive disorder among United States adults increased from USD 210.5 billion in 2010 to USD 333.7 billion in 2019, representing an increase of 58% [4].

Beyond the large economic burden on society, recent advances in medicine have further suggested that the systemic inflammation that accompanies depression may also insidiously trigger dysfunctions in the immune system, thereby predisposing the affected persons to other immune-related diseases, especially rheumatoid arthritis (RA) [5]. Recent evidence from a population-based study shows that those with major depression might double their likelihood of having RA [6]. Crosstalk between depression and RA has been noted, likely resulting from shared underlying pathogenic mechanisms between the brain and the immune system [7]. Individuals with depression were found to have elevated levels of proinflammatory cytokines, such as interleukin (IL)-6, IL-1β, tumor necrosis factor (TNF)-alpha, and C-reactive protein, in both their peripheral blood and cerebrospinal fluid, compared with healthy controls [8]. Through humoral and neural routes, these inflammatory precursors promote articular chondrocyte apoptosis in synovial tissues and the degradation of cartilage and bone via the intracellular signaling pathway, such as Janus kinase-signal transducer and activator of transcription (JAK-STAT) and phosphoinositide 3-kinase/protein kinase B (PI3K/AKT), which, in turn, leads to subsequent bone damage with the expression of matrix metalloproteinase [6,9]. Worse yet, individuals with episodes of depression may face up to nearly a twice higher risk of death than the general population [10]. Given the shared linkage between RA and depression, implementing an alternative complementary treatment to proactively manage subsequent autoimmune disease flare-ups may benefit the well-being of those affected by depression.

At present, the conventional antidepressant drugs include selective serotonin reuptake inhibitors (SSRIs), noradrenaline reuptake inhibitors, agomelatine, bupropion, and vortioxetine [11]. However, a significant proportion of patients taking these medications often experience adverse side effects, including nausea, insomnia, headaches, gastrointestinal symptoms, and sexual dysfunction, leading to notable rates of relapse [12]. The use of Chinese herbal medicines (CHMs), however, might provide an alternative option in treating depression. This manner appears to be long-standing adjunctive therapy, and its success appears to be comparable to that of Western medical treatment in controlling symptoms of depression with fewer side effects [13]. Importantly, these plant-derived compounds have demonstrated immunomodulatory and anti-inflammatory actions that have been shown to inhibit the release and function of cytokines. A previous study reports that rats fed saikosaponin-D (1 mg/kg for 7 days) secreted clearly lower levels of proinflammatory cytokines than did untreated controls [14]. While studies have attempted to elucidate the molecular basis of the antidepressant effects of traditional herbal treatments [15,16], what is at issue now is that no study has explored the long-term effect of CHMs on the prevention of RA onset among patients with depression.

On the basis of the saying that “an ounce of prevention is worth a pound of care”, the application of long-term assessment, based on a large population-based database, should aid in bridging the research gap between CHMs use and the risk of RA and provides the most recent information for healthcare providers to manage these two disorders. This nested patient–control study aimed to address whether adding CHMs to conventional treatment may be helpful in decreasing the risk of RA in persons with depression.

## 2. Results

The analysis included data from 973 patients with RA and 1946 controls with no RA. The corresponding sociodemographic and clinical features are shown in Table 1. The mean age was 50.89 years (SD: 11.36), and nearly 80% were female. The majority of enrollees had the median monthly income level (50.8%) and tended to live in more urbanized areas (54.1%). Pertinent characteristics (age, sex, monthly income, residential area, and comorbidities) did not differ significantly between the patient and control groups.

During the study period, 41.4% of the patients and 51.1% of the controls were taking CHMs. From the multivariate analyses of CHMs use and RA risk, we observed that CHMs users had a lower risk of being diagnosed with RA as compared to the non-CHMs comparators (adjusted ORs: 0.64; 95% CIs: 0.57–0.76). Notably, those using CHMs for more than 3 years had the most striking reduction in RA episodes, followed by those using CHMs for 1–3 years and those using them for less than 1 year, with ORs of 0.39, 0.50, and 0.71, respectively. This finding reveals an exposure–response inverse association between the duration of CHMs use and probability of having RA (Table 2). Importantly, the therapeutic efficacy still persisted even when stratified by sex and age (Table 3).

**Figure 1:**
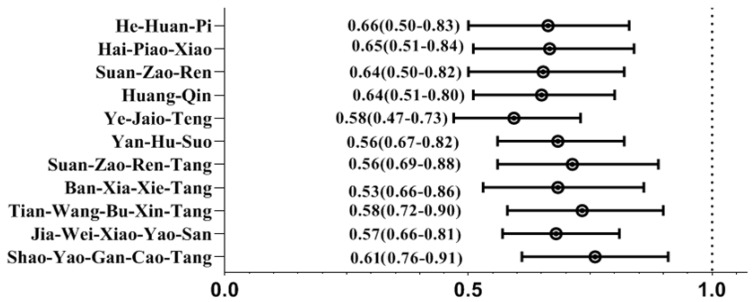
Risk of RA by multivariate conditional logistic regression among the commonly used herbs.

We noted that CHMs administration provided the greatest therapeutic benefit if initiated within the first 2 years of depression onset (adjusted ORs: 0.52; 95% CIs: 0.33–0.79). The benefit of CHMs use in reducing the RA risk decreases with the delayed initiation of CHMs treatment (Table 4). Additionally, of the most commonly used CHMs herbs for treating depression, several formulae correlated to a lower likelihood of RA: Jia-Wei-Xiao-Yao-San; Tian-Wang-Bu-*Xin*-Dan; Ban-Xia-Xie-Xin-Tang; Shao-Yao-Gan-Cao-Tang; Suan-Zao-Ren-Tang; Yan-Hu-Suo; Ye-Jiao-Teng; Huang-Qin; Suan-Zao-Ren; Hai-Piao-Xiao; and He-Huan-Pi (Figure 1). *W*e also summarized the elements and corresponding functions of the commonly used herbal products listed in this work (Table 5).

## 3. Discussion

Using the presently available antidepressant medications, decreasing the risk of RA onset among patients with depression seems to be a formidable task. Given the dire adverse effects of concomitant depression and RA, early preventive measures are greatly needed. This study is the first to observe an association between CHMs use and a lower subsequent risk of RA among patients with depression. We found that integrating CHMs into conventional care decreased the incidence of RA among patients with depression. This benefit was greater with a longer period of CHMs use, independent of age and sex. Specifically, we observed a 61% decrease in RA onset among patients using CHMs with conventional antidepressants for more than three years. In addition, our findings suggest that initiating CHMs use within the first 2 years after depression onset may have the greatest therapeutic effect. While head-to-head comparisons with matched relatives of target patients remain to be employed, the current findings support previous findings that CHMs are beneficial for treating rheumatic disorders [17].

Another important aim of our work was to identify the specific CHMs constituents that contribute to a reduction in the RA risk in the target population. We observed that Jia-Wei-Xiao-Yao-San and Ban-Xia-Xie-Xin-Tang were the most frequently used multi-herb agents for treating depression; both reduced the likelihood of incident RA by nearly 40%. In clinical practice, patients with depression often experience abdominal gastrointestinal symptoms such as nausea, cramps, and bloating [18]. These herbs are often prescribed to treat the neuropsychological disorders and their accompanying symptoms. The proposed mechanisms by which these herbs protect against RA primarily involve the regulation of the inflammatory responses. One systematic review reported that Jia-Wei-Xiao-Yao-San, commonly prescribed to treat Graves’ disease, exerted anti-inflammatory effects that diminished the apoptosis of thyroid follicular cells through the IL-17 signaling pathway [19]. Ban-Xia-Xie-Xin-Tang is proven to alleviate gastrointestinal motility disorders and also exhibits immunomodulatory and anti-inflammatory properties [20]. A recent animal study further proved that this herb markedly downregulated the expression of the inflammatory factors IL-1β and TNF-alpha by inhibiting the NF-κB signaling pathway [21]. Shao-Yao-Gan-Cao-Tan, another herbal remedy, mainly lessens the abdominal pain caused by acute gastroenteritis [22]. In addition, this herb has been shown to inhibit the secretion of inflammatory mediators in rats with polycystic ovary syndrome by blocking the TLR4/NF-*κ*B signaling pathway [23]. This pathway plays an important role in the generation of inflammatory cytokines, thereby potentially provoking a range of other autoimmune disorders [24].

We found that the uses of CHMs prescriptions containing Suan-Zao-Ren-Tang and Tian-Wang-Bu-Xin-Dan displayed promising clinical effects on RA prevention. In clinical practice, these formulas are often prescribed to help patients alleviate restlessness or stress, especially for sleep disturbances that accompany other chronic diseases [25,26]. Several pharmacological studies have reported that Suan-Zao-Ren-Tang exerted anti-convulsant activity, nourished the blood, protected the cardiovascular system, and improved immunity [27]. A study in mice reported that *Z.* jujuba, one of the major constituents of this herb, notably decreased the levels of serum proinflammatory markers, including TNF-alpha, nitric oxide, IL-1β, and IL-6 [28], thereby diminishing the predisposition to RA. Another study showed that Tian-Wang-Bu-Xin-Dan extract decreased the levels of inflammatory factors in rats by regulating the nuclear factor kappa beta (NF-κB) and MAPK signaling cascades [16], both of which are known to play an essential role in the pathogenesis of rheumatic disorders [29].

Of the single-herb products used to treat depression and its accompanying symptoms, we found that Yan-Hu-Suo and Hai-Piao-Xiao significantly lowered the likelihood of RA among our target population. The underlying mechanisms by which these herbs act to arrest RA onset may involve the modulation of inflammatory mediators. For example, Berberine is a known active component of Yan-Hu-Suo that has analgesic properties. In addition to relieving pain, this extract inhibits IL-1β, IL-6, and TNF-alpha RNA expression and decreases their protein expression by attenuating NF-κB phosphorylation [30]. Similarly, our findings indicate that Hai-Piao-Xiao may be beneficial for preventing RA onset. Another randomized control trial showed that chitosan, a major constituent of this herb, greatly increased peripheral sensory nerve regeneration through its profound antioxidant properties [31]. A novel meta-analysis centering on experiments using chitosan-injected mice found that chitosan exerts a substantial anti-inflammatory effect [32]. Taken together, the findings from these earlier studies present evidence that may explain why the use of these remedies was found to decrease the risk of RA in our study.

A positive correlation was also observed between the prescriptions of Ye-Jiao-Teng and Huang-Qin and a reduced risk of RA. A cell culture experiment showed that CRPE55IB, an anthraquinone compound from Polygonum multiflorum thunb, greatly inhibited the inflammatory response in LPS-stimulated microglia by upregulating the AMPK/Nrf2 pathway [33]. This compound alleviates neuroinflammation caused by neurodegeneration and also activates the antioxidant activity of superoxide dismutase and glutathione peroxidase [34]. Mounting evidence suggests a direct correlation between a breakdown in the glutathione system and autoimmune disease [35]. We observed an association between Huang-Qin use and a lower incidence of RA. In a previous study, this medication was shown to modulate colitis and dose-dependently inhibit serum levels of inflammatory cytokines by regulating the inflammation-associated pathways Ras-PI3K-Akt-HIF-1α and NF-κB [36].

We also observed an association between He-Huan-Pi use and a lower risk of RA. This *herb* has long been recognized to possess a mild sedative effect to treat conditions like anxiety, stress, and depression for a long time [37]. The administration of He-Huan-Pi to mice decreased the mRNA expression and the secretion of IL-6 in LPS-stimulated peritoneal macrophages [38]. Activated macrophages contribute to the inflammatory response by producing nitric oxide, which, upon its release, causes tissue injury and contributes to damage in diseases such as arthritis, diabetes, arteriosclerosis, and cancer [39]. This, in a nutshell, is the reason that this herb may act on a cellular level in preventing RA onset.

Our study is the first to investigate whether the usage of CHMs modifies the chance of RA in patients with depression. Our findings suggest that the administrations of CHMs together with conventional antidepressants could substantially decrease the subsequent probability of RA onset. The NHI database used to establish the study cohort possesses several important merits. The outcome measurement was consistent, and the cohort was selected from records that covered nearly 99% of insured residents in Taiwan. Another advantage is the application *of* longitudinal data, which allowed us to robustly establish temporal sequencing, aiding in the identification of a cause-and-effect relationship. Crucially, given the relatively low incidence of RA, the use of longitudinal follow-ups that spanned for over a 10-year period greatly strengthens our confidence in the demonstrated benefits of CHMs use. Finally, our patient–control approach used within an established cohort to evaluate time-dependent drug use differs from the conventional method. We believe that the validity of the findings of the present study will be efficient in clinical practice and may be comparable to those derived from randomized, placebo-controlled trials.

Despite being a pioneering work, the findings from this study must be interpreted with the realization of several shortages. First, the study findings are based on medical records in an administrative healthcare database. Such data might be subject to errors in patient status assignments that can result in misclassification bias, causing biased estimates of the conditions being studied. Nevertheless, the NHI regularly checks medical charts to ensure the accuracy of claims and medical charge data. We believe that patient misclassification would be independent of any of the measured variables, so that point estimates would be biased, if at all, in a more conservative direction. Second, some *factors* that might influence the predisposition to RA, such as lifestyle behaviors, family history, and underlying biochemical deficiencies, were not available in the claims-based database. Thus, residual confounding factors could have affected the results. Embedding a randomized clinical trial into an ongoing larger cohort of patients with depression is needed to elucidate the potential mechanisms of CHMs herbs on the prevention of RA onset. Third, information on the severity of depression was not available in this database, and failure to consider this factor might affect the accuracy of the findings. To this end, we carried out a sensitivity analysis in which we only included depressed patients with no comorbidities. This re-analysis showed that those receiving CHMs therapy still had a 35% lower risk of RA than that of their matched counterparts (adjusted ORs: 0.65; 95% CIs: 0.54–0.75), indicating that the observed results were unlikely to have been prejudiced by the hidden disease severity.

## 4. Materials and Methods

### 4.1. Data Source

The data used in this study were derived from the National Health Insurance Research Database (NHIRD) that was released by the Ministry of Health and Welfare. Taiwan began its National Health Insurance program in 1995 to finance healthcare and lessen the financial gap for its residents. More than 25 million individuals are enrolled in the program, representing 99% of the entire population of Taiwan [40]. The database includes records of all medical benefit claims for ambulatory and inpatient care and is used extensively in epidemiological studies. In this database, all medical diagnoses are documented according to the International Classification of Diseases, Ninth Revision, Clinical Modification (ICD-9-CM) codes. Because the data used were scrambled before being released for research use, information identifying an individual, at any level, cannot be retrieved from the database. The Institutional Review Board of the Buddhist Dalin Tzu Chi Hospital also confirmed that this study was exempt from full review by the institution because only de-identified secondary data were used (No. B11004025-1).

### 4.2. Study Cohort

We identified subjects 20–70 years of age with new-onset depression episodes in the NHIRD and focused primarily on those who had ever sought relevant healthcare services for depression between 2002 and 2010. The diagnosis of depression was regarded as valid if the ICD-9-CM code was documented at least twice in outpatient clinic records within 1 year or at least once during hospitalization (ICD-9-CM codes 296.2, 296.3, 300.4, or 311). The date of the first diagnosis of depression was considered the cohort entry date. To adhere to established research procedures, we excluded patients who had been followed for less than one year and those who had incomplete data (*n* = 108). Patients diagnosed with depression after an RA episode were excluded to ensure the appropriate temporal direction of the depression and incident RA (*n* = 417). The remaining patients comprised the cohort and were followed up until the earliest occurrence of RA, withdrawal from the NHI program, or the end of 2013, whichever occurred first.

### 4.3. Identification of Patient and Control Groups

**Figure 2:**
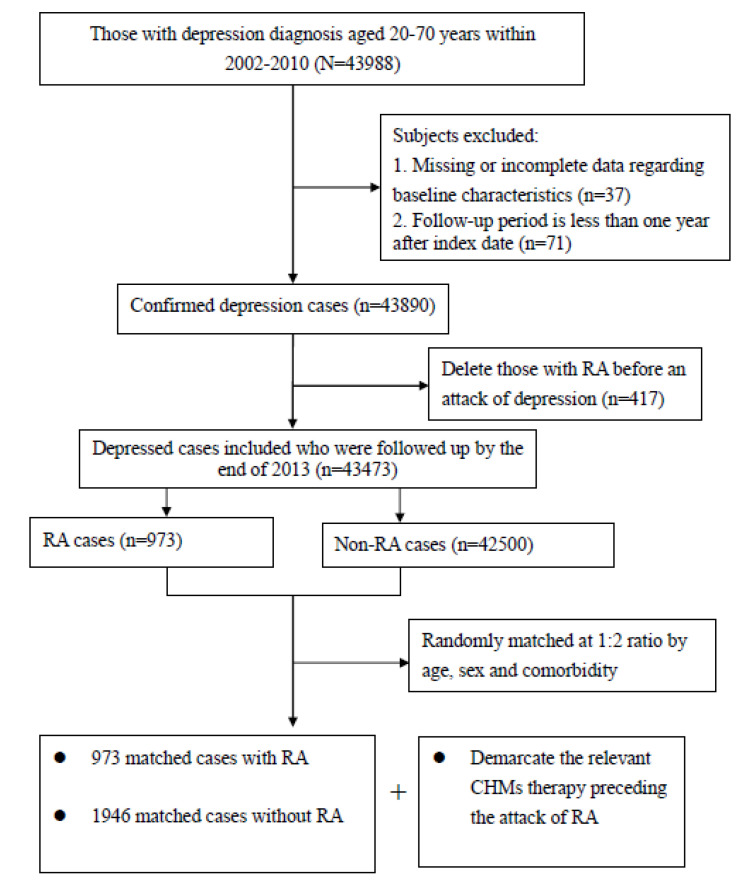
Flowchart of patient selection.

The primary outcome for this study was the emergence of RA, occurring between 2003 and 2013 and after the onset of depression. We applied the catastrophic illness registry to verify the patient RA status. Only those patients certified as having a catastrophic illness with the ICD-9-CM code of 714.0 were deemed as suffering from RA. In Taiwan, insured residents with major diseases (e.g., cancer, autoimmune disease, chronic mental disease, end-stage renal failure) can apply for a catastrophic illness certificate to exempt them from co-payment. Those who met the aforesaid criteria were defined as having a diagnosis of RA. The first medical visit due to RA was viewed as the index date. Afterwards, each patient was randomly matched to two controls without the diagnosis of RA by age, sex, and comorbidities (Figure 2). An index date was then assigned to the controls according to the RA diagnosis date of the matched patient, thereby ensuring the same observational timeframe for the two groups.

### 4.4. Identification of CHMs Use

CHMs use was obtained from the medical records of visits to Chinese medicine practitioners. *It* was investigated during the time period from the cohort entry to the index date. Patients were designated as CHMs users if they underwent relevant CHMs treatments for depression, or its symptoms, for more than 30 days. The CHMs users were further divided into three subgroups according to the number of days for which CHMs were prescribed within the study timeframe. This method enabled us to further investigate the exposure–response risk of RA according to the number of days of CHMs use.

### 4.5. Definition of Covariates

Based on former reports [41,42], the covariates analyzed included sex, age, individual monthly salary, urbanization level of residential area, and previous medical comorbidities. The premium payment category was used as a substitute for family income and was then transformed to ordinal indicators as the 25th, 50th, or 75th percentile. Furthermore, a commonly adopted rule recommended by Liu and colleagues was used to separate the urbanization level of the enrollees’ residential areas into three groups [43]. This indicator was established based on several dimensions, such as the population density per square kilometer, proportion of persons with an educational level of college or above, proportion of the population aged 65 years or older, percentage of labor force work in agriculture, and number of clinicians per 100,000 inhabitants. Medical comorbidity was defined as a condition diagnosed at least once on an inpatient, or twice on an outpatient, claimed one year preceding the cohort entry date. The Charlson–Deyo comorbidity index (CCI), a method for evaluating the number and severity of 17 pre-defined comorbid conditions (range: 1–6), was used to assess the comorbidity impacts [44].

### 4.6. Statistical Modeling

Applications of SAS software for Windows version 9.4 and SPSS 22.0 started the statistical analysis in this study. Descriptive statistics, including means, standard deviations (SD), frequencies, and percentages, were applied to analyze the patients’ characteristics at baseline. Between-group differences were examined using the standardized difference score. As compared to conventional tests, this approach is not influenced by the sample size and is often recommended for comparing baseline covariates in clinical studies. A standardized difference score of less than 0.1 indicated that the difference between two groups did not reach statistical significance [45]. Conditional logistic regression was then executed to estimate odds ratios (ORs) with 95% confidence intervals (CIs) to determine the relation between CHMs use and subsequent RA episodes. Crude ORs based on a simple model, including only CHMs use, and the adjusted ORs for a full model with all participants’ background variables were then determined. For all statistical analyses, the significance level was set at 0.05 for a two-tailed *p*-value.

## 5. Conclusions

The serious consequences of chronic inflammation in patients with depression necessitates the application of available therapeutic modalities, especially *in* the association of CHMs use with the subsequent risk of RA. Findings of the present study show that patients who had CHMs integrated into their conventional antidepressant care experienced a significantly lower risk of RA. Furthermore, this therapeutic effect appears to be dose-dependent, with long-standing CHMs use further lessening the likelihood of subsequent RA. Apart from shedding light on the evidence regarding the long-term effects of CHMs use on the prevention of RA episodes, this study paves the way for further in vivo studies on specific herbal prescriptions to assess their effects on preventing or treating autoimmune disorders. As far as clinical practice is concerned, it is important that psychiatrists are aware of the detrimental implications of rheumatic diseases due to the shared pathogenesis between these two disorders. To this end, taking proactive approaches to regularly monitor depressed patients’ tissue lining the joints is highly recommended. Not only that, an early diagnostic surveillance and opportune therapeutic intervention via one multidisciplinary musculoskeletal team may be considered to improve the clinical outcomes among such groups.

## Figures and Tables

**Table 1:** Demographic data and selected comorbidities of patients and controls.

Variables	Number (%)	Patients	Controls	Standardized Difference
		*n* = 973 (%)	*n* = 1946 (%)	
**Age (years)**				0.006
≤ 50	1515 (51.9)	507 (52.1)	1008 (51.8)	
> 50	1404 (48.7)	466 (47.9)	938 (48.2)	
Mean	50.89 ± 11.36	50.72 ± 11.38	51.02 ± 11.35	0.03
Sex				0.02
Male	612 (21.0)	204 (21.0)	408 (21.0)	
Female	2307 (79.0)	769 (79.0)	1538 (79.0)	
Monthly income				0.02
25th percentile	1334 (45.7)	451 (46.4)	883 (45.4)	
50th percentile	1483 (50.8)	488 (50.2)	995 (51.1)	
75th percentile	102 (3.5)	34 (3.5)	68 (3.5)	
Residential area				0.0005
Urban	1580 (54.1)	527 (54.2)	1053 (54.1)	
Suburban	333 (11.4)	112 (11.5)	221 (11.4)	
Rural	1006 (34.5)	334 (34.3)	672 (34.5)	
CCI	5.01 ± 9.77	4.94 ± 9.07	5.04 ± 11.35	0.009

Abbreviations: SD, standard deviation; CCI, Charlson–Deyo Comorbidity Index.

**Table 2:** Relationship between use of CHMs and risk of RA onset during the study timeframe.

CHMs Exposure	Patients				Crude ORs (95% CIs)	Adjusted ORs* (95% CIs)
	Patients *n* = 973		Controls *n* = 1946			
Non-CHMs users	570	58.6	952	48.9	1	1
CHMs users	403	41.4	994	51.1	0.67 (0.58–0.77)	0.64 (0.57–0.76)
Group 1 (31 days–1 year)	351	36.1	805	41.4	0.72 (0.61–0.85)	0.71 (0.61–0.84)
Group 2 (1–3 years)	37	3.8	131	6.7	0.49 (0.32–0.68)	0.50 (0.32–0.67)
Group 3 (3 years or more)	15	1.5	58	3.0	0.40 (0.24–0.76)	0.39 (0.23–0.74)

* Adjusted for potential confounders, including age, sex, residential area, monthly income, and CCI. Abbreviations: CHMs, Chinese herbal medicines; ORs, odds ratios; CIs, confidence intervals; CCI, Charlson–Deyo Comorbidity Index.

**Table 3:** Age- and sex-specific risk of RA among patients with depression with and without CHMs use.

	RA Patients, *n* (%)	Crude OR (95% CI)	Adjusted OR (95% CI)
Sex			
Male	255 (20.6)	0.52 (0.35–0.76)	0.52 (0.37–0.78) *
Female	985 (79.4)	0.32 (0.27–0.36)	0.31 (0.25–0.36) *
Age (years)			
≤ 50	641 (49.8)	0.31 (0.25–0.40)	0.31 (0.24–0.37) **
> 50	599 (50.2)	0.39 (0.37–0.50)	0.36 (0.31–0.51) **

* Model adjusted for age, residential area, monthly income, and CCI. ** Model adjusted for sex, residential area, monthly income, and CCI. Abbreviations: RA, rheumatoid arthritis; CHMs, Chinese herbal medicines; ORs, odds ratios; CIs, confidence intervals.

**Table 4:** Risk of RA in patients with depression with and without CHMs use according to time of CHMs initiation.

Follow-Up Duration	Non-CHMs Users		CHMs Users	
	Adjusted ORs	95% CIs	Adjusted ORs *	95% CIs
< 2 years	1	reference	0.52	0.33–0.79
2–5 years	1	reference	0.73	0.51–1.05
> 5 years	1	reference	1.21	0.86–1.59

* Model adjusted for age, sex, urbanization level, monthly income, and comorbidities. Abbreviations: CHMs, Chinese herbal medicines; ORs, odds ratios; CIs, confidence intervals.

**Table 5:** The ingredient herbs contained in the most-used single-herb and multi-herb products among participants.

Chinese Herbal Product	Ingredients	Functional Classification
Single–Herb Products		
Hai–Piao–Xiao	Endoconcha Sepiae Os Sepiae seu Sepiellae	Controls acidity, harmonizes the stomach, and alleviates pain
Ye–Jiao–Teng	Caulis Polygoni Multiflori	Nourishes the heart and liver blood and expels wind in the collaterals to stop itch and treat skin disorders
Mu–Li	Ostrea gigas Thunb.	Antacid traditional medicines have also has been used for treating bone fractures
Yan–Hu–Suo	Corydalis yanhusuo	Used to treat Qi stagnation, blood stasis, chest pain, abdominal pain, amenorrhea, dysmenorrhea, and postpartum stasis
Bei–Mu	Fritillariae Thunbergii Bulbus	Eliminates phlegm by cooling, moistens lungs to arrest cough, and removes stasis to reduce swelling
Da–Huang	Rheum officinale.	Addresses constipation and other inflammatory issues in the colon, liver, gallbladder, stomach, and reproductive organs
Suan–Zao–Ren	Ziziphi Spinosae Semen	Sedative hypnosis, anti-anxiety, antidepressant, anticancer, anti-inflammatory, anti-Alzheimer’s disease (AD) effects
Bai–Zhi	Angelica dahurica	Anti-inflammation, anti-tumor, antioxidation, and analgesic activity and antiviral and anti-microbial effects on the cardiovascular system, neuroprotective function, hepatoprotective activity, skin diseases, and so on
He–Huan–Pi	Albizia julibrissin Durazz.	Used for depression and anxiety treatment
Huang–Qin	Scutellaria baicalensis	Used as an adjuvant therapy for inflammation, diabetes, hypertension, and different kinds of cancers and virus-related diseases
Multi–Herb Products		
Jia–Wei–Xiao–Yao–San	Bupleurum root, Chinese Angelica root, white peony root, White Atractylodes rhizome, Poria, licorice root, moutan bark, gardenia fruit, mint herb, ginger	Used to treat functional dyspepsia
Suan–Zao–Ren–Tang	Ziziphi Spinosae Semen, Poria, Chuanxiong Rhizoma, Anemarrhenae Rhizoma, and Glycyrrhizae Radix Et Rhizoma	Used to treat insomnia and anxiety
Ban–Xia–Xie–Xin–Tang	Pinellia ternate, Makino, Panax ginseng, Zingiber officinale Roscoe, Coptis chinensis Franch., Scutellaria baicalensis Georgi, Ziziphus jujuba Mill	Used to treat metabolic diseases, such as nonalcohol fatty liver disease, diabetes mellitus, and obesity
Chuan–Xiong–Cha–Tiao–San	Radix Chuanxiong, Herba Schizonepetae, Radix Saposhnikoviae, Rhizoma et Radix Notopterygii, Radix et Rhizoma Glycyrrhizae, Radix Angelicae Dahuricae, Herba Menthae, Radix et Rhizoma Asari	Used to treat headaches caused by externally contracted wind pathogens
Shu–Jing–Huo–Xie–Tang	Tang-kuei root, white peony root, Corydalis root, Chin-chiu, Cnidium root, raw rehmannia root, peach kernel, Hoelen fungus, Atractylodes root, citrus peel, Notopterygium root, fragrant angelica, Scabrous gentiana root, Fang feng root, Achyranthes root, ginger root, Chinese licorice root	Clears heat, cools the blood, nourishes Yin, and generates fluids; breaks up blood stasis and invigorates blood circulation; strongly dries dampness, tonifies the spleen, induces sweating, and expels wind-dampness; promotes urination and leaches out dampness
Tian–Wang–Bu–Xin–Dan	Ginseng Radix et Rhizoma, Poria, Scrophulariae Radix, Salviae Miltiorrhizae Radix et Rhizoma, Platycodonis Radix, Polygalae Radix), Angelicae Sinensis Radix), Schisandrae Chinensis Fructus, Ophiopogonis Radix, Asparagi Radix, Platycladi Semen), Ziziphi Spinosae Semen, Rehmanniae Radix	Enriches Yin; clears heat; nourishes blood; calms the mind
Ge–Gen–Tang	Kudzu root (Pueraria lobata), Cassia twig (Cinnamomum cassia), fresh ginger rhizome (Zingiber officinale), Chinese peony root without bark (Paeonia lactiflora), Chinese licorice root and rhizome (Glycyrrhiza uralensis), jujube fruit (Ziziphus jujuba), Notopterygium root and rhizome (Notopterygium incisum), pubescent angelica root (Angelica pubescens), Sichuan lovage rhizome (Ligusticum chuanxiong), Bupleurum root (Bupleurum chinense)	Chinese herbs for upper respiratory tract infection, cervical myositis, tendinitis of the shoulder, lymphadenitis, and cerebrovascular disease
Gan–Mai–Da–Zao–Tang	Blighted wheat, licorice, jujube	Cerebral microcirculatory regulation, mood stabilization, and the alleviation of impatience
Xiang–Sha–Liu–Jun–Zi–Tang	Ginseng, hoelen, atractylodes macrocephala koidz, liquorice root, pinelliae tuber, pericarpium citri, common ginger, jujube, amomum villosum costusroot.	Invigorate the spleen, harmonize the stomach, and regulate the Qi flow to relieve pain
Shao–Yao–Gan–Cao–Tang	Paeonia Lactiflora, Glycyrrhiza Uralensis	Treat spleen and liver blood deficiency.

## Data Availability

This study obtained data from the National Health Insurance Research Database provided by the Bureau of National Health Insurance, managed by the Department of Health and Welfare, Taiwan. As per the release of the “Personal Information Protection Act” law, the relevant data cannot be publicly obtained. Requisition for the usage of datasets should be directed to the Bureau of National Health Insurance and the corresponding author.
